# The relationship between different ventricular rate control levels and cardiac remodeling in early persistent atrial fibrillation: a prospective cohort study

**DOI:** 10.3389/fcvm.2024.1447907

**Published:** 2025-01-02

**Authors:** Yongrong Liu, Jun Liu, Dan Wang

**Affiliations:** ^1^ Department of Cardiovascular Medicine, People’s Hospital of Chongqing Hechuan, Chongqing, China; ^2^ Department of Cardiovascular Medicine, First Affiliated Hospital of Zhengzhou University, Zhengzhou, China

**Keywords:** ventricular rate, ventricular rate control levels, cardiac remodeling, atrial fibrillation, persistent atrial fibrillation, early persistent atrial fibrillation, different ventricular rate, different ventricular rate control

## Abstract

**Background:**

Atrial fibrillation (AF) is a prevalent cardiac arrhythmia, with ventricular rate control being a critical therapeutic target. However, the optimal range for ventricular rate control remains unclear. Additionally, the relationship between different levels of ventricular rate control and cardiac remodeling in patients with atrial fibrillation remains unclear.

**Objective:**

This study aims to explore the relationship between different levels of heart rate control and cardiac remodeling in patients with early persistent atrial fibrillation.

**Methods:**

A bi-center prospective cohort study was conducted, enrolling patients with newly diagnosed persistent AF and rapid ventricular rates, yet with a normal cardiac size, from March 2019–May 2020 at the people's hospital of Chongqing Hechuan and the First Affiliated Hospital of Zhengzhou University. Patients were divided into four groups based on their average ventricular rate levels from 24 h Holter monitoring: Group I (40 ≤ average rate < 60 bpm), Group II (60 ≤ average rate <80 bpm), Group III (80 ≤ average rate < 100 bpm), and Group IV (average rate ≥ 100 bpm).The study tracked changes in left atrial diameter (LAD), left ventricular end-diastolic diameter(LVEDD),left ventricular ejection fraction(LVEF), and the severity of mitral regurgitation over one year.

**Results:**

A total of 764 patients were enrolled. We found that there were no significant differences in cardiac remodeling among the groups of patients before the observation. However, after one-year follow-up observation, there were significant differences in the degree of cardiac remodeling among the groups (*p* < 0.001). Specifically, the severity of cardiac remodeling, including LVEDD, LAD, LVEF, and mitral regurgitation, showed the following trend: Group II < Group I < Group III < Group IV. Further regression analysis indicated that body mass index (BMI) might be related to changes in LAD. Additionally, the use of digoxin could affect changes in left ventricular ejection fraction. At the same time, the use of diltiazem, bisoprolol, as well as factors like hypertension, coronary artery disease, smoking, diabetes, and chronic obstructive pulmonary disease, might be closely associated with the worsening of mitral regurgitation.

**Conclusion:**

This study shows that in early persistent AF patients, different levels of heart rate control are related to varying degrees of cardiac remodeling. These results suggest that maintaining an average ventricular rate within the range of 60–80 beats per minute may be associated with milder cardiac remodeling. On the other hand, an average heart rate greater than 100 bpm appears to be associated with the most severe cardiac remodeling.

**Registration Number:**

ChiCTR2400079978; Registered 17 January 2024–Retrospectively registered: https://www.chictr.org.cn/showproj.html?proj=198684.

## Introduction

Atrial fibrillation (AF) is the most common cardiac arrhythmia encountered in clinical settings. With the global aging population, its prevalence increases annually. Although recent studies suggest that rhythm management strategies like catheter ablation might outperform ventricular rate control strategies such as pharmacotherapy (1), catheter ablation also presents limitations. These include high treatment costs, a notable risk of recurrence, and surgery-related risks, which significantly challenge healthcare budgets in developing countries.

During AF, normal sinus *P* waves are replaced by disorganized and rapid fibrillation waves, leading to the complete absence of the A wave in the trans-mitral flow (2). This results in a loss of atrial booster pump function, drastically reducing ventricular filling volume during diastole, lowering stroke volume, and severely compromising cardiac function (3, 4). Without effective ventricular rate control in AF patients, the rapid and irregular ventricular rate can further deteriorate ventricular diastolic function, potentially leading to severe clinical complications such as heart failure (5).

The pathophysiology of AF involves both electrical and structural remodeling of the heart. cardiac remodeling in AF includes a series of changes such as enlargement of the left atrium, dilation of the left ventricle, reduction in left ventricular ejection fraction (LVEF), and development of mitral regurgitation (6). These changes are not only markers of disease progression but also lead to worsened clinical outcomes in AF patients.

The management of AF mainly focuses on two strategies: rhythm control and rate control. Ventricular rate control, in particular, is crucial in the treatment of AF as it aims to normalize the heart rate, thereby alleviating symptoms and preventing tachycardia-induced cardiomyopathy (7). However, the impact of different levels of ventricular rate control on cardiac remodeling remains unclear. Although clinical trials have shown that high ventricular rates in AF patients are associated with increased risks of heart failure and death (8, 9), there is still controversy over the optimal level of ventricular rate control.

Therefore, this study aims to fill this gap by exploring the relationship between different levels of ventricular rate control and cardiac remodeling in patients with early persistent atrial fibrillation. Through a prospective cohort study, we will assess the role of ventricular rate control in the management of atrial fibrillation, with a particular focus on its effects on changes in left ventricular function and structure. The study will provide new insights into how different levels of heart rate control influence the progression of cardiac remodeling. The findings suggest that attention should be paid to the level of ventricular rate control during the treatment of atrial fibrillation, which may help mitigate cardiac remodeling and offer better clinical guidance.

## Method and material

### Study population

This study is a dual-center prospective observational cohort study from Chongqing and Zhengzhou, China (ChiCTR2400079978). The study included 1,016 patients who were first diagnosed with AF in the outpatient department between March 2019 and May 2020.

The first phase involved using outpatient routine electrocardiograms for preliminary screening of patients, showing newly diagnosed AF. Then, the first round of follow-up was carried out, recording baseline data of all patients, as well as pre-measurements of left atrial diameter (LAD), left ventricular end-diastolic diameter (LVEDD), mitral regurgitation, and LVEF grouping values. In the second phase, after one month of ventricular rate control treatment, a standard electrocardiogram (ECG) and a Holter monitor test were performed again to determine whether the patient had persistent AF. If both the standard ECG and Holter monitor results indicated AF, the patient was presumed to have persistent AF. Patients who met the criteria were divided into four groups based on the average ventricular rate levels reported from 24-hour dynamic electrocardiography monitoring: Group I with an average ventricular rate of 40≤ and <60 bpm, Group II with 60≤ and <80 bpm, Group III with 80≤ and <100 bpm, and Group IV with an average ventricular rate ≥100 bpm. Stratified random sampling was used to select eligible patients from each group until the sample size in each reached 200, after which no further sampling was done for that group. The third phase included a follow-up one year later to collect data on LAD, LVEDD, mitral regurgitation, and LVEF for each group.

***Inclusion Criteria***: (1) Age between 50 and 75 years; (2)Patients seen in the cardiology clinic and emergency departments, with a standard twelve-lead ECG showing the initial diagnosis of AF (newly diagnosed AF); (3) Accompanied by rapid ventricular rate (ventricular rate ≥100 bpm); (4) Normal size of the left atrium and left ventricle (LAD ≤35 mm, LVEDD ≤50 mm); (5) LVEF ranging from 50% to 65%. ***Exclusion Criteria:*** (1) Valvular heart disease: Refers to any degree of stenosis of the mitral and aortic valves, as well as patients with artificial heart valves. (2) Concurrent hyperthyroidism or diagnosed hyperthyroid heart disease; (3) Tachy-brady syndrome; (4) Myocardial disease; (5) Planned restoration of sinus rhythm; (6) Transition to sinus rhythm during the follow-up period; (7) 24-hour Holter monitor suggesting long RR intervals >5 s. All enrolled patients were required to sign an informed consent form and received approval from the ethics committee. A total of 764 patients were included in the final analysis.

### Sample size calculation

For this study, sample size was calculated using hypothesis testing methods. With an alpha of 0.05, beta of 0.1, and an effect size of 0.3, a minimum of 173 patients were required in each group. Considering potential loss to follow-up and data quality issues, the sample size was increased by 10%, totaling 800 patients. Based on the grouping proportions for different ventricular rate control levels, the sample sizes for each group were: Group I (40 ≤ average ventricular rate < 60 bpm, *n* = 200), Group II (60 ≤ average ventricular rate < 80 bpm, *n* = 200), Group III (80 ≤ average ventricular rate < 100 bpm, *n* = 200), and Group IV (average ventricular rate ≥ 100 bpm, *n* = 200).

### Data collection

Baseline data of eligible patients were collected, including gender, age, hypertension, diabetes, smoking, body mass index (BMI), alcohol consumption, coronary artery disease, and medications such as diltiazem, beta-blockers (Due to the centralized drug procurement policies led by the Chinese government, bisoprolol was the only beta-blocker available at our two centers), digoxin, and non-vitamin K antagonist oral anticoagulants (NOAC) (Due to the centralized drug procurement policy led by the Chinese government, rivaroxaban was the only NOAC available in our two centers).Data on cardiac echocardiography were collected from eligible patients at their initial visit (pre-observation) and one year later (post-observation), including LAD, LVEDD, LVEF, and mitral regurgitation.

### Diagnosis of early persistent AF

AF was initially diagnosed based on a 12-lead electrocardiogram in the outpatient setting, which showed the absence of sinus *P* waves, replaced by fine fibrillatory f waves and an absolutely irregular RR interval. Moreover, after one month of ventricular rate control treatment, a standard ECG and a Holter monitor test were performed again to determine whether the patient had persistent AF. If both the standard ECG and Holter monitor results indicated AF, the patient was presumed to have persistent AF.

### Measurement of mitral regurgitation severity

Mitral regurgitation was semi-quantitatively graded based on the length of the regurgitant jet into the left atrium. Mild regurgitation: the regurgitant jet reaching the lower third of the left atrium at the annular level. Moderate regurgitation: the regurgitant jet reaching one-third to two-thirds of the left atrium. Severe regurgitation: the regurgitant jet reaching the top of the atrium or extending beyond two-thirds into the pulmonary veins.

### Measurement of left atrial diameter

In the parasternal long-axis view of the left ventricle, M-mode echocardiography was used to measure the anteroposterior diameter of the left atrium, from the anterior edge of the aortic posterior wall to the posterior edge of the left atrium, perpendicular to the aortic root.

### Measurement of left ventricular end-diastolic diameter

The LVEDD was measured using M-mode echocardiography in the parasternal long-axis view, with the M-mode cursor placed at the tips of the mitral valve, ensuring the cursor was perpendicular to the interventricular septum, with the boundary defined by the interface between blood and tissue.

### Measurement of left ventricular ejection fraction

The LVEDD and left ventricular end-systolic volumes were measured in apical four chamber and two chamber views. Calculations from both views were used to calculate the average LVEF using Simpson's method.

### Statistical analysis

SPSS 26.0 was used for statistical analysis. Chi-square test and one-way ANOVA were used for comparisons of categorical and numerical variables between groups. Multivariate linear regression models were employed to assess relationships between changes in LAD, LVEDD, LVEF, and different groupings, with covariates selected based on literature. Potential confounders included age, gender, BMI, smoking and alcohol use, medications such as diltiazem, bisoprolol, digoxin, and conditions like hypertension, coronary artery disease, diabetes, and chronic obstructive pulmonary disease (COPD). Subsequently, we employed non-parametric testing methods of the Kruskal-Wallis test to compare these variables among different groups. Regarding the change in mitral regurgitation, a regression analysis was performed using logistic regression. Patients with increased severity of mitral regurgitation observation were marked as 1, and those without increased severity were marked as 0. All analyses used a 95% confidence level, with *p* < 0.05 considered statistically significant.

## Sensitivity analysis

To analyze the sensitivity of the four regression models mentioned above, we attempted to change the values of the input variables to explore the corresponding changes in the dependent variables. Specifically, representative values for control variables were calculated (for continuous variables, such as age and BMI, the median was used; for discrete variables, like smoking and hypertension, the mode was used), representing a typical sample. For this study, the independent variable (group membership) was changed, and the predicted outcomes of the models for this sample's change in LAD, LVEDD, LVEF, and severity of mitral regurgitation aggravation were plotted, under different group conditions but with other control variables remaining constant.

## Results

**Figure 1:**
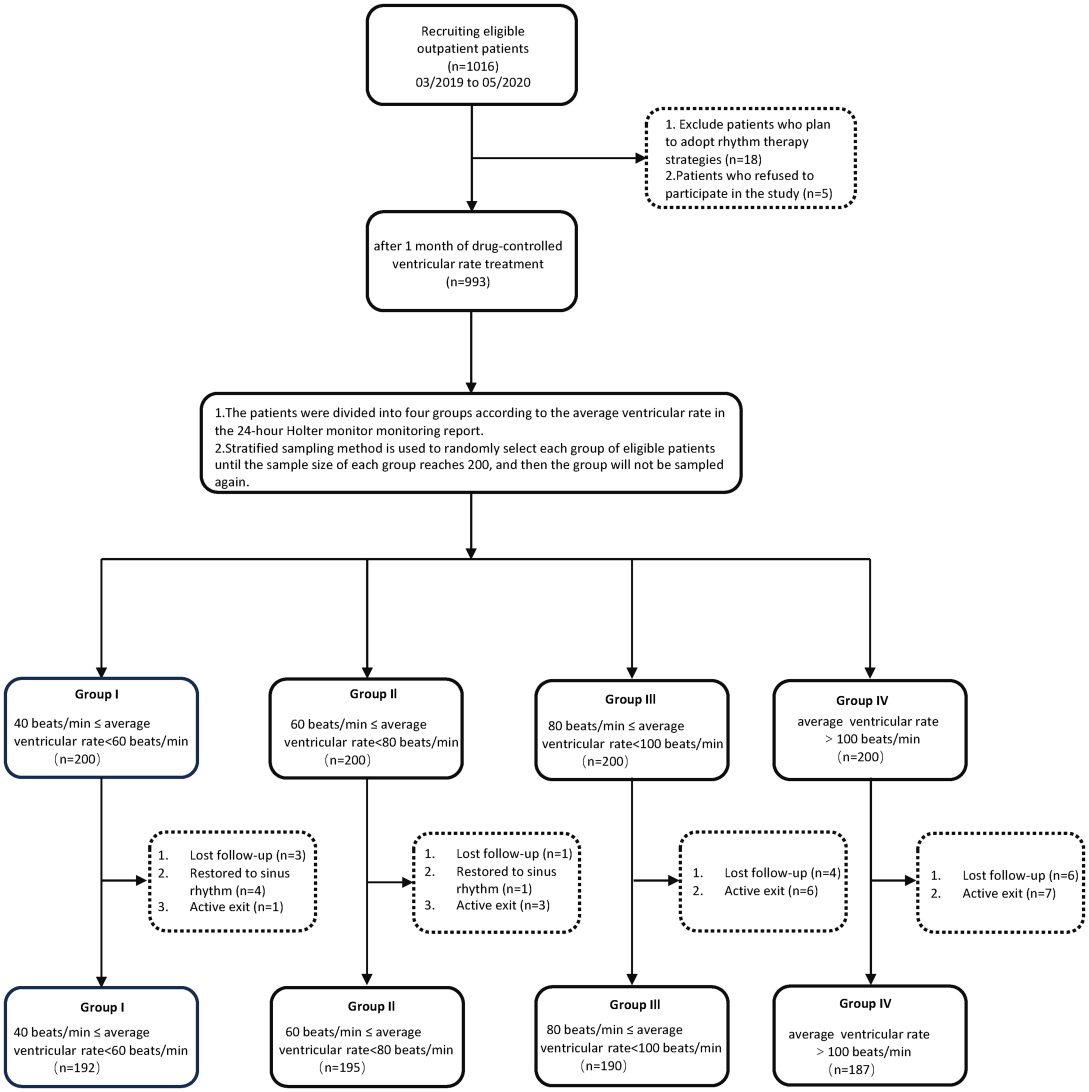
Flow chart depicting the study design for examining the effects of ventricular rate control on cardiac remodeling in patients with early persistent AF.

Initially, 1,016 eligible patients were included in the study. Five patients refused to participate. 18 patients who planned to adopt a rhythm control strategy were excluded (During the initial screening, we had already excluded patients who were planned to restore sinus rhythm based on the exclusion criteria. However, during the one-month period of medication for heart rate control, 18 patients changed their treatment choice to rhythm control. Therefore, these 18 patients were excluded.). After one month of pharmacological treatment to control the ventricular rate, 993 patients were included. Based on the average ventricular rate results from 24 h dynamic electrocardiograms, patients were divided into four groups using stratified random sampling. Patients were randomly selected from each group until the sample size reached 200, after which no further sampling was conducted for that group. After one year, follow-up data showed that in Group I, 3 patients were lost to follow-up, 4 reverted to sinus rhythm, and 1 withdrew, resulting in 192 remaining. In Group II, 1 was lost to follow-up, 1 reverted to sinus rhythm, and 3 withdrew, resulting in 195 remaining. In Group III, 4 were lost to follow-up and 6 withdrew, leaving 190. In Group IV, 6 were lost to follow-up and 7 withdrew, leaving 187 remaining (Figure 1).

A significant difference in age was observed across the four groups, with Group I averaging 62.27 years, Group II 65.07 years, Group III 67.97 years, and Group IV 62.15 years, indicating a statistically significant difference (Table 1; *p* < 0.001). However, gender distribution across these groups did not demonstrate a notable difference, with a nearly balanced representation of males and females in each group (Table 1; *p* = 0.549). Regarding medication usage, diltiazem showed a varied utilization across groups, with the highest in Group II (89.74%) and the lowest in Group IV (52.94%), signifying a significant difference (Table 1; *p* < 0.001). Bisoprolol usage also displayed notable differences, with near-universal usage in Group I (100%) and slightly lesser in other groups, which was statistically significant (Table 1; *p* = 0.002). In contrast, the use of digoxin and rivaroxaban was more evenly distributed among the groups, not showing significant differences. The study also noted a significant variation in the prevalence of hypertension across groups, with the highest prevalence in Group III (71.58%) and the lowest in Group IV (Table 1; 57.75%; *p* = 0.041). Smoking habits were significantly different among the groups, with the highest prevalence in Group III (54.74%) and the lowest in Group I (Table 1; 39.58%; *p* = 0.015). The prevalence of coronary heart disease, diabetes, and COPD also varied among groups but did not reach statistical significance. Lastly, a significant difference was noted in BMI values across the groups, indicating a potential link with the studied cardiac conditions (Table 1).

**Table 1:** Characteristics of the participants.

Variable	Group I	Group II	Group III	Group IV	*χ*²/F	*P*
Age (years)	62.27 ± 4.79	65.07 ± 4.08	67.97 ± 3.31	62.15 ± 3.86	204.667	<0.001
Male	99 (51.56%)	107 (54.87%)	100 (52.63%)	89 (47.59%)	2.116	0.549
Female	93 (48.44%)	88 (45.13%)	90 (47.37%)	98 (52.41%)		
Diltiazem	163 (84.90%)	175 (89.74%)	136 (71.58%)	99 (52.94%)	82.350	<0.001
Bisoprolol	192 (100.00%)	191 (97.95%)	180 (94.74%)	175 (93.58%)	15.015	0.002
Digoxin	23 (11.98%)	19 (9.74%)	26 (13.68%)	24 (12.83%)	1.568	0.667
Rivaroxaban	192 (100.00%)	195 (100.00%)	190 (100.00%)	187 (100.00%)	0.000	1.000
Hypertension	119 (61.98%)	126 (64.62%)	136 (71.58%)	108 (57.75%)	8.276	0.041
Smoke	76 (39.58%)	91 (46.67%)	104 (54.74%)	78 (41.71%)	10.420	0.015
Drink	28 (14.58%)	19 (9.74%)	22 (11.58%)	31 (16.58%)	4.677	0.197
Coronary disease	76 (39.58%)	68 (34.87%)	57 (30.00%)	52 (27.81%)	7.109	0.068
Diabetes	90 (46.88%)	74 (37.95%)	96 (50.53%)	82 (43.85%)	6.624	0.085
BMI	24.10 ± 3.22	25.30 ± 2.86	25.89 ± 2.06	26.20 ± 2.52	46.339	<0.001
COPD	24 (12.50%)	33 (16.92%)	28(14.74%)	46(24.60%)	11.012	0.012

COPD, chronic obstructive pulmonary disease; BMI, body mass index.

**Figure 2:**
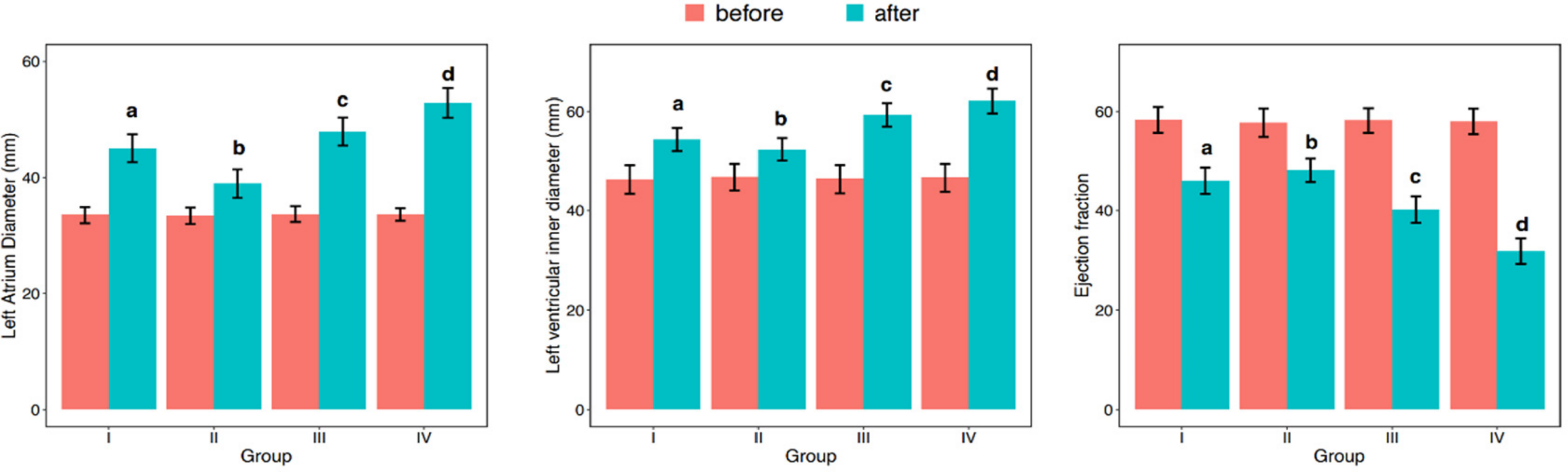
Differences among groups in LAD (left atrial diameter), LVEDD, (left ventricular end-diastolic diameter), and LVEF (left ventricular ejection fraction) before and after one-year follow-up observation.

Table 2 presents the differences in LAD, LVEDD, and LVEF before and after one-year follow-up observation across different groups. Initially,irrespective of the observation period, none of the variables –LAD, LVEDD, or LVEF—followed a normal distribution (Table 2; Figure 2; *p* < 0.001). Consequently, to compare these variables among different groups, the study employed non-parametric testing methods, specifically the Kruskal-Wallis test. According to the table, before observation, there were no significant differences between the groups in terms of LAD (*p* = 0.175), LVEDD (*p* = 0.319), and LVEF (*p* = 0.098). However, after one-year follow-up, significant differences were observed in all three variables –LAD (*p* < 0.001), LVEDD (*p* < 0.001), and LVEF (*p* < 0.001)—among the four groups. Additionally, pairwise comparisons revealed that these differences occurred between any two groups. And, all parameters of each group of patients showed varying degrees of deterioration in the clinical setting (Figure 2).

**Table 2:** Differences in LAD, LVEDD, and LVEF before and after one-year follow-up observation.

	Group I	Group II	Group III	Group IV	Test value	*p*
LAD (before)	33.48 ± 1.40	33.37 ± 1.43	33.67 ± 1.36	33.60 ± 1.09	4.953	0.175
LVEDD (before)	46.26 ± 2.86	46.74 ± 2.71	46.34 ± 2.86	46.60 ± 2.83	3.513	0.319
LVEF (before)	58.31 ± 2.59	57.73 ± 2.87	58.16 ± 2.49	57.99 ± 2.60	6.290	0.098
LAD (after)	45.06 ± 2.40^a^	38.93 ± 2.47^b^	47.95 ± 2.40^c^	52.88 ± 2.55^d^	624.396	<0.001
LVEDD (after)	54.34 ± 2.35^a^	52.25 ± 2.27^b^	59.30 ± 2.35^c^	62.06 ± 2.46^d^	571.221	<0.001
LVEF (after)	46.01 ± 2.66^a^	48.13 ± 2.37^b^	40.17 ± 2.66^c^	31.83 ± 2.54^d^	626.097	<0.001

LAD, left atrial diameter; LVEDD, left ventricular end-diastolic diameter; LVEF, left ventricular ejection fraction.

Different letters (a, b, c, d) represent significant differences between groups based on Tukey's HSD test (*p* < 0.05).

**Figure 3:**
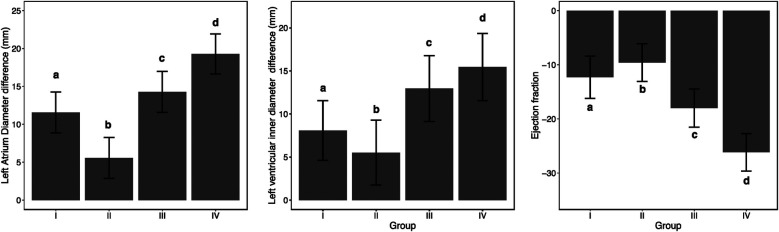
Analysis of differences in LAD (left atrial diameter), LVEDD (left ventricular end-diastolic diameter), and LVEF (left ventricular ejection fraction) between different groups before and after one-year follow-up observation.

Table 3 shows the differences in changes in LAD, LVEDD, and LVEF before and after one-year follow-up observation among different groups. The table indicates that there were significant differences in the changes of LAD (*p* < 0.001), LVEDD (*p* < 0.001), and LVEF (*p* < 0.001) before and after one-year follow-up observation among the four groups. Additionally, pairwise comparisons revealed that these differences occurred between any two groups (Figure 3).

**Table 3:** Analysis of differences in LAD, LVEDD, and LVEF between different groups before and after one-year follow-up observation.

	Group I	Group II	Group III	Group IV	Test value	*p*
LAD difference before and after	11.57 ± 2.69^a^	5.56 ± 2.70^b^	14.28 ± 2.70^c^	19.28 ± 2.64^d^	599.083	<0.001
LVEDD difference before and after	8.08 ± 3.46^a^	5.51 ± 3.77^b^	12.96 ± 3.83^c^	15.46 ± 3.90^d^	417.067	<0.001
The difference in LVEF before and after	−12.30 ± 3.92^a^	−9.60 ± 3.50^b^	−17.99 ± 3.53^c^	−26.17 ± 3.46^d^	565.326	<0.001

**Figure 4:**
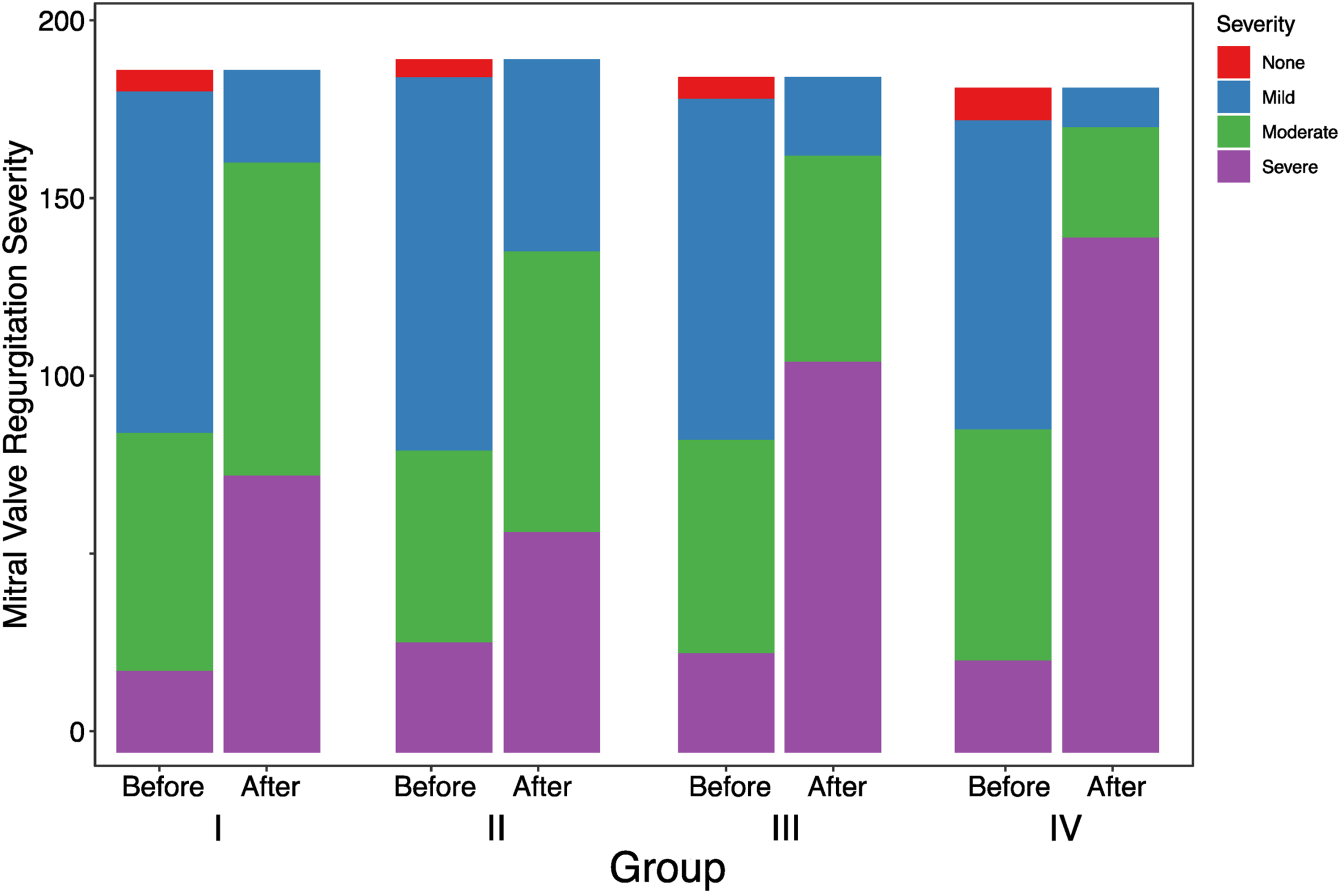
Observation of differences among groups in mitral regurgitation before and after one-year follow-up observation.

Table 4 displays the differences in mitral regurgitation before and after one-year follow-up observation across different groups. Before observation, the distribution of varying degrees of mitral regurgitation showed no significant difference among the groups (*p* = 0.762). However, after one-year follow-up observation, there were significant differences in the distribution of mitral regurgitation among the groups (*p* < 0.001) (Figure 4).

**Table 4:** Observation of differences in mitral regurgitation before and after one-year follow-up observation.

Mitral regurgitation	Level	Group I	Group II	Group III	Group IV	χ²	*p*
	Severe	23	31	28	26	5.779	0.762
Before	Moderate	67	54	60	65		
	Mild	96	105	96	87		
	No	6	5	6	9		
	Severe	78	62	110	145	106.627	<0.001
After	Moderate	88	79	58	31		
	Mild	26	54	22	11		
	No	0	0	0	0		

In conclusion, in our comprehensive analysis of cardiac remodeling under varying heart rate control conditions, we observed differential outcomes across the groups after a one-year follow-up. The data, as detailed in Tables 2, 3, illustrate significant changes in LAD, LVEDD, and LVEF. Notably, Group II, which maintained an average heart rate of 60–80 beats per minute, exhibited the least cardiac remodeling compared to the other groups. This group's modest heart rate control effectively mitigated the progression of left atrial enlargement, LVEDD increase, and LVEF reduction, showcasing a clinically beneficial approach to managing early persistent atrial fibrillation (AF). Pairwise comparisons further confirmed that the degree of deterioration in Group II was significantly lower than in the other groups, where higher average heart rates were correlated with more pronounced cardiac remodeling. This trend was similarly reflected in the changes observed in mitral regurgitation, with Group II showing the least worsening after one year, as illustrated in Table 4 and Figure 4.

We performed multiple linear regression analyses to assess the impact of various groups on changes in LAD, left ventricular end-diastolic diameter (LVEDD), and left ventricular ejection fraction (LVEF), as detailed in Tables 5–7 respectively. The regression models for all parameters were statistically significant. The models explained a substantial proportion of the variance in each parameter (77.9% for LAD, 53% for LVEDD, and 76% for LVEF). The change in LAD followed the pattern: Group II < Group I < Group III < Group IV. Similar patterns were observed for changes in LVEDD and LVEF, with significant differences among the groups. The tables provide a complete breakdown of the regression coefficients and their significance levels. These findings suggest that groups based on varying levels of heart rate control, significantly varied in cardiac remodeling.

**Table 5:** Regression of changes in LAD.

	Regression coefficient	Standard error	*t*	*p*	0.025	0.975
Constant	9.535	1.855	5.141	<0.001	5.894	13.176
Group II	−6.029	0.295	−20.413	<0.001	−6.609	−5.449
Group III	2.461	0.337	7.297	<0.001	1.799	3.124
Group IV	7.470	0.321	23.28	<0.001	6.840	8.100
Age (years)	−0.010	0.024	−0.406	0.685	−0.057	0.038
Gender	−0.028	0.195	−0.144	0.886	−0.41	0.354
Diltiazem	−0.447	0.369	−1.210	0.227	−1.172	0.278
Bisoprolol	0.493	0.567	0.87	0.385	−0.619	1.605
Digoxin	0.541	0.753	0.719	0.472	−0.936	2.019
Hypertension	0.024	0.351	0.070	0.945	−0.665	0.714
Smoke	0.527	0.469	1.123	0.262	−0.394	1.447
Drink	0.034	0.722	0.048	0.962	−1.382	1.451
Coronary disease	−0.209	0.405	−0.517	0.605	−1.004	0.586
Diabetes	0.316	0.474	0.668	0.504	−0.614	1.246
BMI	0.096	0.036	2.642	0.008	0.025	0.167
COPD	−0.910	0.527	−1.726	0.085	−1.945	0.125

COPD, chronic obstructive pulmonary disease; BMI, body mass index.

**Table 6:** Regression of changes in LVEDD.

	Regression coefficient	Standard error	*t*	*p*	0.025	0.975
Constant	7.327	2.603	2.815	0.005	2.218	12.437
Group II	−2.575	0.414	−6.213	<0.001	−3.389	−1.761
Group III	4.931	0.473	10.417	<0.001	4.002	5.860
Group IV	7.444	0.450	16.531	<0.001	6.560	8.328
Age (years)	−0.001	0.034	−0.033	0.974	−0.068	0.065
Gender	−0.210	0.273	−0.770	0.442	−0.746	0.326
Diltiazem	0.040	0.518	0.076	0.939	−0.978	1.057
Bisoprolol	0.695	0.795	0.874	0.382	−0.866	2.256
Digoxin	1.507	1.056	1.427	0.154	−0.566	3.580
Hypertension	0.504	0.493	1.023	0.307	−0.463	1.472
Smoke	−0.610	0.658	−0.927	0.354	−1.901	0.682
Drink	−1.217	1.013	−1.202	0.230	−3.205	0.771
Coronary disease	0.312	0.568	0.550	0.583	−0.803	1.428
Diabetes	−0.217	0.665	−0.326	0.744	−1.522	1.088
BMI	0.002	0.051	0.045	0.964	−0.097	0.102
COPD	0.430	0.740	0.582	0.561	−1.022	1.883

COPD, chronic obstructive pulmonary disease; BMI, body mass index.

**Table 7:** Regression of changes in LVEF.

	Regression coefficient	Standard error	*t*	*p*	0.025	0.975
Constant	−15.557	2.499	−6.225	<0.001	−20.463	−10.651
Group II	2.609	0.398	6.557	<0.001	1.828	3.390
Group III	−6.063	0.454	−13.340	<0.001	−6.955	−5.171
Group IV	−13.709	0.432	−31.707	<0.001	−14.557	−12.860
Age (years)	0.053	0.033	1.614	0.107	−0.011	0.116
Gender	0.027	0.262	0.103	0.918	−0.488	0.542
Diltiazem	−0.209	0.498	−0.420	0.675	−1.186	0.768
Bisoprolol	1.196	0.763	1.567	0.118	−0.302	2.695
Digoxin	2.389	1.014	2.356	0.019	0.398	4.380
Hypertension	−0.389	0.473	−0.822	0.412	−1.318	0.540
Smoke	0.906	0.632	1.434	0.152	−0.334	2.146
Drink	−1.280	0.972	−1.317	0.188	−3.189	0.628
Coronary disease	−0.298	0.546	−0.546	0.585	−1.369	0.773
Diabetes	0.047	0.638	0.074	0.941	−1.206	1.300
BMI	−0.044	0.049	−0.899	0.369	−0.140	0.052
COPD	−0.910	0.710	−1.281	0.201	−2.304	0.484

COPD, chronic obstructive pulmonary disease; BMI, body mass index.

Logistic regression model achieved an R-squared value of 0.702 (Table 8). Significant RCs were found for Group II (*p* < 0.001), Group III (*p* < 0.001), and Group IV (*p* < 0.001). Compared to group I, RC was −4.182, 3.678 and 6.975 for groups II, III and IV respectively. Thus, the proportion of worsened mitral regurgitation is: Group II < Group I < Group III < Group IV.

**Table 8:** Regression of changes in mitral regurgitation.

	Regression coefficient	Standard error	*t*	*p*	0.025	0.975
Constant	−11.92	3.181	−3.748	<0.001	−18.154	−5.686
Group II	−4.182	0.804	−5.2	<0.001	−5.758	−2.605
Group III	3.678	0.716	5.137	<0.001	2.275	5.082
Group IV	6.975	1.07	6.518	<0.001	4.878	9.073
Age (years)	0.042	0.038	1.109	0.267	−0.032	0.117
Gender	0.154	0.308	0.498	0.619	−0.451	0.758
Diltiazem	6.033	0.965	6.252	<0.001	4.142	7.924
Bisoprolol	6.759	1.355	4.989	<0.001	4.103	9.414
Digoxin	−28.099	78,789.19	0.000	1.000	−1,54,452	1,54,395.9
Hypertension	2.725	0.57	4.78	<0.001	1.607	3.842
Smoke	−1.549	0.615	−2.519	0.012	−2.755	−0.344
Drink	−3.004	1.618	−1.857	0.063	−6.176	0.167
Coronary disease	7.439	1.082	6.876	<0.001	5.319	9.56
Diabetes	−6.26	0.987	−6.345	<0.001	−8.194	−4.326
BMI	−0.009	0.056	−0.162	0.871	−0.119	0.101
COPD	−7.514	1.693	−4.438	<0.001	−10.832	−4.195

COPD, chronic obstructive pulmonary disease; BMI, body mass index.

**Figure 5:**
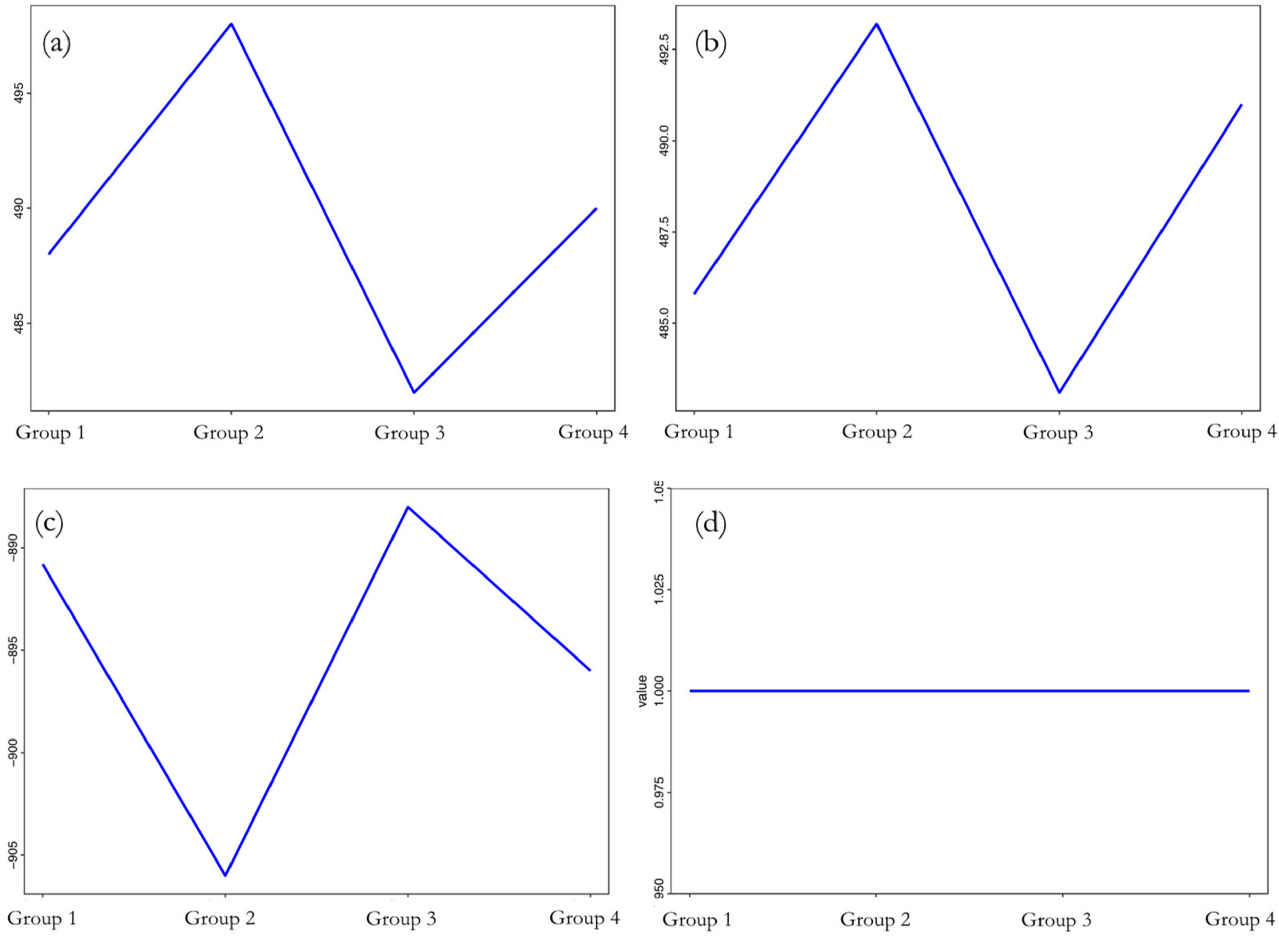
Sensitivity analysis of differences to **(a)** LAD; **(b)** LVEDD; **(c)** LVEF; **(d)** mitral regurgitation before and after one-year follow-up observation.

Figure 5 showed that when the sample's group category changed, there were drastic changes in the differences in LAD, LVEDD, and LVEF before and after one-year follow-up observation. Specifically, when changing from Group I to Group II, both the change in left atrial and LVEDD increased significantly; from Group II to Group III, there was a significant decrease in both; and from Group III to Group IV, these changes increased again. In terms of LVEF, its change also varied significantly with the change in group category. The change in LVEF decreased substantially when changing from Group I to Group II, increased significantly from Group II to Group III, and then slightly decreased from Group III to Group IV. As for the severity of mitral regurgitation aggravation, it was observed that changing the sample's group had almost no effect. Thus, it was evident that the changes in LAD, LVEDD, and LVEF before and after one-year follow-up observation were highly sensitive to changes in group categories. However, the impact of group change on the aggravation of mitral regurgitation was found to be very low.

## Discussion

This study found some differences in cardiac remodeling related to variation in heart rate control in newly diagnosed persistent AF patients. Specifically, the severity of cardiac remodeling, including LVEDD, LAD, LVEF, and mitral regurgitation, showed the following trend: Group II < Group I < Group III < Group IV. Group II performs the best in terms of cardiac remodeling. When the average ventricular rate exceeded 80 beats per minute (bpm), the increase in ventricular rate was associated with a gradual increase in the diameter of the left atrium and left ventricle, indicating a positive correlation between ventricular rate and heart enlargement. At the same time, the LVEF was negatively correlated with the level of ventricular rate; the higher the rate, the poorer the cardiac contractile function. Additionally, if the average ventricular rate exceeded 80 bpm, mitral regurgitation tended to worsen. We observed that the use of diltiazem and bisoprolol may be associated with exacerbation of mitral regurgitation, indicating that the use of these drugs may have specific effects on cardiac remodeling. Although we found that the use of digoxin may affect changes in LVEF, studies have shown that the use of digoxin in patients with atrial fibrillation and heart failure is associated with a higher risk of all-cause mortality, especially in newly used patients, where the risk is more significant (10, 11). This reminds us that the use of digoxin needs to be very cautious when controlling heart rate in patients with AF. The study also found that factors like hypertension, smoking, coronary artery disease, diabetes, and COPD might be linked to worsening mitral regurgitation. The results suggest that the optimal heart rate range for patients with AF might be between an average ventricular rate of 60–80 bpm. This helps alleviate cardiac remodeling, reduces the risk of heart failure, and improves clinical outcomes, which is significantly important for managing AF patients.

Some studies have indicated that atrial enlargement is one of the main mechanisms of AF onset and maintenance (12, 13), closely related to impaired left ventricular diastolic function. Additionally, a higher ventricular rate is also considered a factor contributing to AF (14) and left ventricular remodeling (15). This study offers new insights, for example, past research typically recommended a lenient control strategy for controlling the heart rate in AF, i.e., a resting heart rate below 110 bpm (16). The difference between this study and the RACE II study lies in two main aspects: (1) The assessment method in RACE II study is very limited, only evaluating patients' heart rates at a specific resting or active moment, which cannot fully represent the daily heart rate levels of patients with persistent AF. This study combines dynamic ECG monitoring and uses average ventricular rate as an indicator, which can better demonstrate the ventricular rate control level and its effectiveness in AF patients, helping clinicians understand the patients' daily heart rate control levels. (2) The results of the RACE II study indicate that lenient heart rate control is not inferior to strict ventricular rate control in terms of cardioembolic stroke and mortality, which may give the impression that heart rate control is not important. However, the results of this study emphasize the importance of heart rate control and suggest that heart rate should be maintained at a reasonable level, such as using dynamic ECG for evaluation, keeping the average ventricular rate within the range of 60–80 beats per minute. Our study's results indicate that a ventricular rate ≥100 bpm is associated with the most severe cardiac remodeling, consistent with recent research by Westergaad-Lucas Malta and colleagues, where a ventricular rate ≥100 bpm was linked with a higher risk of new-onset heart failure (17). Our study differs in that it finds that different levels of ventricular rate control in patients with early persistent AF have varying impacts on cardiac remodeling. This suggests that in the treatment of AF, it is also important to lower the heart rate while maintaining it at an appropriate level to achieve better treatment outcomes. We also found that maintaining an average ventricular rate of 60–80 beats per minute might be optimal, as indicated by measurements such as LAD, LVEDD, and LVEF, this range is associated with less cardiac enlargement and deterioration of ventricular function. Moreover, our study emphasizes the importance of using dynamic electrocardiography to assess the heart rate level of patients with persistent AF, as continuous monitoring can more accurately reflect the patient's all-day heart rate level.

However, this study has some limitations. First, the sample size is relatively small, including only newly diagnosed patients with fast ventricular rates and normal heart size with persistent AF, which may not represent all AF patients. Secondly, the follow-up period is only one year, preventing observation of longer-term cardiac remodeling changes. Moreover, the study used dynamic electrocardiographic monitoring as a tool to measure average ventricular rate, but the accuracy of dynamic electrocardiographic monitoring might be affected by the duration patients wear the monitor. Future research could expand in several directions: first, by increasing the sample size, adding multicenter studies, and analyzing patients of different races, ages, and genders. Secondly, by extending the follow-up period to observe longer-term changes in cardiac remodeling and related clinical outcomes. Third, by using various tools such as smart wearable devices to measure average ventricular rate and comparing their accuracy and reliability (18). Fourth, although cohort studies can provide some evidence for causal inference, they are still generally limited in terms of causality and cannot draw definitive conclusions about causal relationships. Fifth, despite our efforts to include major known confounding factors (such as age, gender, hypertension, and diabetes) in our regression model, we acknowledge that there may still be other potential confounding factors not accounted for, which could affect the results. Moreover, the choice of medication might be influenced by the doctor's preferences and the specific circumstances of the patient, which limits the general applicability of our results. Finally, further exploration of the impact of different treatment approaches on cardiac remodeling, including pharmacotherapy, ablation, and pacing, should be conducted. This study can help to better understand cardiac remodeling in AF patients and provide more accurate guidance for clinical practice.

Despite the availability of many new technologies and methods to restore and maintain sinus rhythm and improve patient prognosis, AF cannot be completely cured at present. Even after restoring sinus rhythm, there is still a possibility of recurrence. The surgical costs of AF radiofrequency ablation or pulsed field ablation are a huge challenge for developing countries like China, which may significantly affect patients' choices of treatment methods and strategies. On the other hand, the relatively inexpensive drug rhythm control strategy has certain drug side effects and requires more frequent follow-ups, which may greatly impact patient compliance with treatment. Ventricular rate control strategy has the advantages of being economical, convenient, and highly compliant, making it a more widely accepted treatment option for patients in developing countries like China.

In summary, this prospective observational study provides new evidence for the relationship between different levels of ventricular rate control and cardiac remodeling in patients with early persistent atrial fibrillation. The research highlights the importance of rate control in slowing down cardiac remodeling and improving clinical outcomes. However, it's worth noting that for early persistent AF patients, especially those with normal atrial and ventricular sizes, rhythm control therapy remains the most effective treatment strategy. When patients opt for rate control due to various reasons, clinicians should consider the effects of different heart rate levels on cardiac remodeling, especially on the sizes of the left atrium and ventricle. Based on the study findings, it is recommended that clinicians aim to maintain the average ventricular rate between 60 and 80 bpm when planning treatment for patients with early persistent AF. Furthermore, for patients with difficulty to achieve optimal rate control, alternative rate controlling strategies like pacemaker implantation followed by atrioventricular node ablation should be considered. Else, AF management strategy should be switched to rhythm control strategy if appropriate.

## Data Availability

The datasets presented in this study can be found in online repositories. The names of the repository/repositories and accession number(s) can be found in the article/Supplementary Material.

## References

[1] Kirchhof P Camm AJ Goette A Brandes A Eckardt L Elvan A Early rhythm-control therapy in patients with atrial fibrillation. N Engl J Med. (2020) 383(14):1305–16. 10.1056/NEJMoa2019422 32865375

[2] Alam M Thorstrand C. Left ventricular function in patients with atrial fibrillation before and after cardioversion. Am J Cardiol. (1992) 69(6):694–6. 10.1016/0002-9149(92)90169-Y 1536123

[3] Leonard JJ Shaver J Thompson M. Left atrial transport function. Trans Am Clin Climatol Assoc. (1981) 92:133–41. 7281408 PMC2279521

[4] Cui Q Wang H Zhang W Wang H Sun X Zhang Y Enhanced left atrial reservoir, increased conduit, and weakened booster pump function in hypertensive patients with paroxysmal atrial fibrillation. Hypertens Res. (2008) 31(3):395–400. 10.1291/hypres.31.395 18497457

[5] Pandey A Kim S Moore C Thomas L Gersh B Allen L Predictors and prognostic implications of incident heart failure in patients with prevalent atrial fibrillation. JACC Heart Fail. (2017) 5(1):44–52. 10.1016/j.jchf.2016.09.016 28034376

[6] Loring Z Clare RM Hofmann P Chiswell K Vemulapalli S Piccini J. Natural history of echocardiographic changes in atrial fibrillation: a case-controlled study of longitudinal remodeling. Heart Rhythm. (2024) 21(1):6–15. 10.1016/j.hrthm.2023.09.010 37717612 PMC10842857

[7] Al-Khatib SM Allen LaPointe NM Chatterjee R Crowley MJ Dupre ME Kong DF Rate-and rhythm-control therapies in patients with atrial fibrillation: a systematic review. Ann Intern Med. (2014) 160(11):760–73. 10.7326/M13-1467 24887617

[8] Nerheim P Birger-Botkin S Piracha L Olshansky B. Heart failure and sudden death in patients with tachycardia-induced cardiomyopathy and recurrent tachycardia. Circulation. (2004) 110(3):247–52. 10.1161/01.CIR.0000135472.28234.CC 15226218

[9] Andrade JG Roy D Wyse DG Tardif JC Talajic M Leduc H Heart rate and adverse outcomes in patients with atrial fibrillation: a combined AFFIRM and AF-CHF substudy. Heart Rhythm. (2016) 13(1):54–61. 10.1016/j.hrthm.2015.08.028 26299677

[10] Chao TF Liu CJ Tuan TC Chen SJ Wang KL Lin YJ Rate-control treatment and mortality in atrial fibrillation. Circulation. (2015) 132(17):1604–12. 10.1161/CIRCULATIONAHA.114.013709 26384160

[11] Vamos M Erath JW Benz AP Lopes RD Hohnloser SH. Meta-analysis of effects of digoxin on survival in patients with atrial fibrillation or heart failure: an update. Am J Cardiol. (2019) 123(1):69–74. 10.1016/j.amjcard.2018.09.036 30539748

[12] Fornengo C Antolini M Frea S Gallo C Grosso Marra W Morello M Prediction of atrial fibrillation recurrence after cardioversion in patients with left-atrial dilation. Eur Heart J Cardiovasc Imaging. (2015) 16(3):335–41. 10.1093/ehjci/jeu193 25274966

[13] Suzuki H Takeishi Y. Inducibility of atrial fibrillation caused by acute increase of atrial pressure in rat diseased heart with chronic atrial dilation. Int Heart J. (2012) 53(4):257–60. 10.1536/ihj.53.257 22878806

[14] Liu X Guo N Zhu W Zhou Q Liu M Chen C Resting heart rate and the risk of atrial fibrillation a dose-response analysis of cohort studies. Int Heart J. (2019) 60(4):805–11. 10.1536/ihj.18-470 31204373

[15] Goette A Lendeckel U. Tachycardia-induced remodeling: atria and ventricles take a different route. Cardiovasc Res. (2004) 63(2):194–5. 10.1016/j.cardiores.2004.05.005 15249176

[16] Van Gelder IC Groenveld HF Crijns HJ Tuininga YS Tijssen JG Alings AM Lenient versus strict rate control in patients with atrial fibrillation. N Engl J Med. (2010) 362(15):1363–73. 10.1056/NEJMoa1001337 20231232

[17] Westergaard LM Alhakak A Rørth R Fosbøl EL Kristensen SL Svendsen JH Ventricular rate in atrial fibrillation and the risk of heart failure and death. Europace. (2023) 25(5):euad088. 10.1093/europace/euad088 37083042 PMC10228534

[18] Yao Y Guo Y Lip GY Lane DA Chen Y Wang L The effects of implementing a mobile health–technology supported pathway on atrial fibrillation–related adverse events among patients with multimorbidity: the mAFA-II randomized clinical trial. JAMA Network Open. (2021) 4(12):e2140071. 10.1001/jamanetworkopen.2021.40071 34932104 PMC8693229

